# Supplementary material for: Are carbon emissions trading and green financial instruments synergistic? -Comprehensive quantitative research based on content analysis
Source: PLoS One. 2024 Mar 7;19(3):e0298601. doi: 10.1371/journal.pone.0298601 (PMC10919601; doi:10.1371/journal.pone.0298601)
Supplement: S1 Data — (ZIP) [file pone.0298601.s001.zip › Data/地级市Z_F工作报告.zip!/┴╔─■/╕╖╨┬╩╨/└┤╫╘╣1⁄2╓┌║┼í╛┬φ┐╦╩2╛▌═°í┐_2010.docx]

各位代表：

　　现在，我代表市政府向大会报告工作，请予审议，并请市政协各位委员和其他列席同志提出意见。

　　一、2009 年工作回顾

　　2009年是不寻常的一年。面对金融危机和特大旱灾的严峻挑战，在市委的正确领导下，全市各族人民团结一心，迎难而上，奋力拼搏，经济社会保持良好的发展势头，“突破阜新”取得显著成效，转型振兴迈出坚实步伐。

　　预计实现地区生产总值270亿元，增长15.2%，增幅高于全省平均水平；全社会固定资产投资240 亿元，增长70.6%，增幅居全省首位；地方财政一般预算收入18.6亿元，增长27.3%，增幅高于全省10 个百分点；直接利用外资6412 万美元，增长24.1%；城市居民人均可支配收入11170 元，增长10.4%；农村居民人均纯收入5535 元，增长10%；万元地区生产总值能耗下降4.8%。

　　（一）“三大基地”和重点产业集群建设取得突破性进展

　　坚持以项目为先，集中精力推进重大项目建设。新开工投资千万元以上项目363个，其中，亿元以上项目76 个。竣工投产投资千万元以上项目77 个，其中，亿元以上项目15个。

　　“三大基地”建设取得新成效。煤化工基地建设稳步推进，备受瞩目的大唐阜新煤制气项目前期工作基本完成，即将开工建设。巴新铁路项目完成投资19亿元，重点控制工程进展迅速。新型能源基地建设实现重大突破，风电项目完成投资48.2 亿元，并网发电72.5 万千瓦，330万千瓦项目签约落地。食品及农产品加工基地不断发展壮大，产值增长28%。

　　重点产业集群发展迅速。引进项目169个，总投资124亿元。液压产业集群实现跨越式发展，引进项目突破100 个，产值实现翻番。现代皮革产业集群奠定良好基础，8个项目开工建设。家具研发制造产业集群扎实推进，24 个项目开工，10 个项目竣工投产。铸造产业集群快速发展，7 个项目投产，产值增长50%。氟化工产业集群产值增长75%。我市被确定为国家液压装备产业化基地、全国首家“承接转移· 中国制革示范基地”、中国北方家具研发制造基地和国家特色铸造产业集群试点市。

　　（二）工业经济实现较快发展

　　采取一系列应对金融危机、促进企业发展的政策措施，工业经济在逆境中保持快速增长势头。规模以上工业增加值76 亿元，增长19%；实现利润5 亿元，同比增盈3.4亿元。83户停产企业相继恢复生产。工业用电量增幅居全省第四位。

　　重点骨干企业进一步发展壮大，规模以上企业新增92 户，达到395户；销售收入超亿元企业新增5 户，达到45户。阜矿集团产业结构调整迈出新步伐，非煤产业比重达到36.9%，玄武岩连续纤维、膨润土开发利用等项目开工建设。阜新发电公司实现减亏3.6亿元，运行质量进一步提高。太克液压、华通管道等39 户企业销售收入增长50%以上；阜新双汇、大金公司等16 户企业上缴税金超过千万元。德尔公司年产汽车动力转向泵120 万台，居全国同行业之首。

　　实施工业技改项目230 个，完成投资80亿元，增长1倍。力迅年产400套风电一体化控制器等23个亿元以上项目开工，超懿热电锅炉改造等16 个亿元以上项目竣工。制定循环经济发展规划和粉煤灰综合利用管理办法，深博粉煤灰综合利用等一批循环经济项目竣工投产。“三沟”品牌荣获中国驰名商标。

　　县区经济快速发展、贡献突出。预计县区生产总值、固定资产投资、财政一般预算收入分别增长18%、80%和30%，均高于全市平均水平。大力发展民营经济，支持中小企业做大做强，民营经济增加值突破百亿元，增长26%。

　　（三）社会主义新农村建设迈出新步伐

　　面对严重旱情，全市上下奋起抗灾自救，实现大灾之年不减收。

　　设施农业突破发展，新建保护地8 万亩，是上年的2.7倍。花生种植面积145 万亩，成为全国花生产业的重点地区之一。畜牧业加快发展，新建畜禽标准化小区502个，生猪、奶业、肉牛、肉鸡等规模和产业化水平进一步提升。新建都市农业项目77个。全年无重大动物疫情发生。

　　林业生态建设积极推进。人工造林和封山育林93 万亩，增长14.5%，完成辽西北边界防护林及高速公路两侧绿化带建设任务。集体林权制度配套改革顺利实施，国有林场改革全面启动。阜、彰两县分别被授予全国林业科技示范县和森林经营采伐试点县。

　　农村水利基础设施建设不断加强。新增灌溉面积4.6万亩，河道生态工程完成9.1万亩，2座中型水库除险加固工程量完成过半。继续推进机械化保护性耕作工程，百万亩地块抗旱效果明显提高。投入粮食综合直补、保护地建设等强农惠农资金7.7亿元，增长57.1%。

　　（四）服务业发展水平不断提高

　　把现代服务业放在突出的战略位置来抓，服务业实现了较快发展。社会消费品零售总额126.5 亿元，增长18.1%。

　　商贸流通业进一步发展。大商集团对新天地广场进行改造提升，阜新兴隆百货正式运营。海州庙商业步行街、新邱区商业步行街一期即将投入使用，市花卉市场等一批专业市场开工建设，鑫维玛瑙宝石城正式开业。“万村千乡”市场工程、“新网工程”覆盖全部行政村。全面开展“家电下乡”工作，补贴销售家电产品4.5万台。早餐工程网点达到31个，方便了市民生活。交通货运量增长13.4%。

　　房地产业快速健康发展。新开工面积242.5万平方米，其中，商品房面积176.5 万平方米，增长88.2%；工业厂房面积66万平方米，创历史最好水平。建筑业产值60 亿元，增长45%。

旅游业发展迅速，总收入实现翻番。海州露天国家矿山公园正式开园，瑞应寺法相学院落成，高山子风电景区成为旅游热点，地热产业开发正式启动。

　　会展业蓬勃发展，成功举办玛瑙博览会、花生博览会、敖包文化节和蒸汽机车摄影节。

　　全市新增贷款65亿元，创历史新高。市商业银行更名为阜新银行，对6 家城市信用社进行整体收购，跨区域经营获得批准。新增小额贷款公司12家、担保公司3家，激活民间资本9.3亿元。阜蒙县村镇银行正式运营。城投债发行上报国家待批。

　　（五）对外开放取得新进展

　　聚焦“三大基地”和重点产业集群，突出主题概念，成功组织赴日韩、港台、新加坡及深圳、温州等7 次大型招商活动，招商引资到位88 亿元，增长54%。华润、神华、中广核、龙源等8户央企落户阜新，尤尼深风机制造、鲁花花生油加工等一批知名品牌项目成功签约，投资8 亿元的富国皮革、投资5.5亿元的德裕制革、投资9 亿元的协合风电设备制造等一批项目开工建设，日本住林木业项目建成投产。

　　出口创汇实现1 亿美元，增幅居全省第二位。对外经济合作取得新进展，意大利援助的市老年康复医院投入使用，日本援助的华东医院项目竣工、苍土乡卫生院项目资金到位。外事、侨务在对外开放中发挥了积极作用。

　　（六）城乡建设水平全面提升

　　城市总体规划修编工作启动，“三位一体”规划深入实施。“ 玉龙新城”开发面积达到100 万平方米，城市发展空间进一步扩大。经营城市取得显著成效，土地纯收益增长1.4 倍。

　　基础设施建设实现重大突破。全市人民瞩目、时速350 公里的京沈客运专线项目获国家批复。沈彰铁路、锦阜铁路扩能改造项目前期工作扎实推进。彰武至通辽、阜新至盘锦高速公路开工建设。城乡道路投资9.6 亿元，增长1.6 倍。人民大街北等3个高速公路出口路及阜铁高速公路阜新站竣工通车。玉龙北路、东新路、北新路和东风路西段等建成通车，玉龙西路、南环路和迎宾大街北段等开工建设。完成国省干道大中修182公里。新建通村油路600 公里，提前一年实现所有行政村通油路。

　　城市功能进一步完善。引白水源日供水能力达到15万吨，供水管网改造二期工程完成17.5公里，“三环制”供热改造工程基本完成。垃圾处理厂二期开工建设。投资7亿元对294公里电网实施改造。3G通信网络正式开通。实施小区物业分类管理，物业管理得到加强。

　　城乡环境明显改善。新增城市绿化覆盖面积174 万平方米，增幅居全省第三位。细河补水能力得到提高，九营子河新建5 座滚水坝。对53个2万平方米以上老旧小区进行综合整治，新建城市公厕17 座，完成农村改厕5000 余座。对29 条街路进行改造，城市中心区基本消灭无灯路。更新公交车辆18台，延伸3条公交线路。落实节能减排责任制，加大城市烟尘整治力度，取缔市区高污染燃料设施240 台，环境空气质量达到二级以上标准天数320天。中心城镇建设步伐加快，产业和人口集聚功能进一步增强。

　　（七）民生工程取得明显成效

　　就业再就业工作成效显著。实现实名制就业6 万人，其中，“零就业家庭”保持动态为零，大学毕业生就业率达到94%。完成普惠制培训8.7万人，组织劳务输出30万人次。

　　社会保障水平进一步提高。城镇离退休人员养老金月标准提高117元，城乡最低生活保障月标准分别提高45 元和17 元。优抚对象抚恤金和生活补助标准平均提高15%，免除城乡低保对象基本殡葬服务等费用。60年代精简职工生活补助标准和新农合筹资标准、报销比例得到提高。

　　保障性住房工程积极推进。建设廉租房10万平方米，完成棚户区改造二期补充工程11.7 万平方米，援助农村低保户新建、维修住房2346 户，解决市级以上劳模住房262 户。低保户廉租住房租金补贴制度启动实施。

　　城乡居民生活条件不断改善。补发了义务教育阶段教师津补贴，提高了非义务教育等事业单位人员工资水平。慈善事业积极发展，8240名困难群众得到救助，“蓓蕾生命救助行动”在全省推广。新建5 所区域性中心敬老院，儿童福利院综合楼投入使用。55 个社区“十分钟服务圈”建设达标。对63个重点贫困村实施整村推进扶贫，10 万农村人口实现稳定脱贫。解决10 万农村人口饮水安全问题。

　　（八）社会事业全面进步

　　科技创新能力不断增强。实施产学研合作项目46个、高新技术研发项目55 个，新增市级研发中心8 家、省级工程技术研发中心8家、省级科技创新企业4家。农业科技特派行动取得较好成效。高新技术产品增加值实现8亿元，增长29%。

　　教育事业优先发展。深入开展新一轮“普九”，教育教学质量进一步提高。新建农村九年制学校8 所，改造维修校舍32.8 万平方米。启动“操场工程”，城区26所中小学新建塑胶操场投入使用。城乡中小学手拉手工程、教师培训提高工程取得实效。职业教育实训基地建设进一步加强，毕业生就业率达到96.3%。彰武林业中等职业技术学校开学，辽工大职业技术学院主体完工，二职专新校开工建设。民办教育进一步发展。

　　人才兴市战略深入实施。引进硕士研究生140名，机关事业单位人才结构得到优化。辽工大成功举办60 周年校庆，服务地方发展取得明显成效。“市长杯”职工技能大赛激发了广大职工练本领、强素质的热情。

文化体育事业蓬勃发展。市图书馆开工建设，工人文化宫改造二期工程主体完工，青少年宫二期投入使用；新建农村综合文化站8个、农家书屋210个。京剧《血胆玛瑙》晋京巡演。宣传我市玛瑙等历史文化的专题片在央视热播。文化信息资源共享工程通过有线电视网络安装5000 户。成功举办第七届市运会。市体育馆主体完工，篮球学院正式招生。创办3个国家级体育后备人才基地，阜新籍运动员在全运会上夺得9枚金牌、亚锦赛上夺得2枚金牌。

　　医疗卫生事业不断进步。疾病预防控制体系和医疗救治体系进一步完善，防控甲型H1N1流感工作取得积极成效。整合医疗资源，市第二人民医院和妇幼保健院搬迁新址。阜矿总医院急救中心、阜蒙县人民医院和彰武县中医院主体完工，中心医院病房楼开工建设。16 所乡镇卫生院、200所甲级村卫生室和20 个乡镇计划生育服务中心建成使用。市新殡仪馆主体完工。

　　群众性精神文明创建活动深入开展，“ 友爱阜新”建设积极推进。开展了丰富多彩的庆祝新中国成立60周年活动。

　　“平安阜新”建设扎实推进。强化社会治安综合治理，严厉打击各类违法犯罪行为，刑事案件发案率下降15.7%，圆满完成国庆安保任务。深入开展“解难题、促和谐”等活动，群众合理诉求大多得到妥善解决。严格落实安全生产责任制，加强隐患排查和整改工作，安全生产形势总体平稳。消防应急指挥综合楼投入使用。

　　国税、地税在促进经济发展、增加财政收入中发挥了重要作用。供电公司提供了安全、优质的电力保障。石油、烟草等中省直单位为全市经济社会发展作出了积极贡献。民族宗教工作得到加强。双拥共建活动蓬勃开展。国有资产监管制度不断完善。第二次全国经济普查顺利完成。县区在地统计工作启动。

　　开展了关爱特困母亲和儿童等活动，妇女儿童工作取得新成绩。物价、人防、地震、档案、民兵预备役等工作取得新进展。

　　（九）政府自身建设进一步加强

　　民主法治建设扎实推进。认真贯彻落实市人大及其常委会的各项决议、决定，自觉接受依法监督。坚持向市政协通报工作制度，自觉接受民主监督。认真听取各民主党派、工商联、无党派人士和各人民团体的意见，民主决策渠道进一步拓宽。办理人大代表建议153 件、政协提案167 件，办结率、满意率均达到100%。强化行政执法监督，依法行政能力进一步增强。大力推进政务公开，市民知情权得到有效保障。

　　服务型政府建设取得积极进展。全面推行并联审批、全程代理制，行政审批效率明显提高。电子政务综合网络平台投入使用，169 家单位实现网络互联。通过市长公开电话、市长信箱及行风热线等渠道，及时解决市民反映的热点、难点问题。在全市164个社区建立市长公开电话联络员制度，进一步密切政府与群众联系。以政府诚信为主导的信用体系建设取得积极成效。

　　政府工作作风不断改进。坚持深入调查研究、现场办公，促进困难和问题的解决。加强机关作风纪律建设，加大行政效能监察力度，政府执行力进一步提高。强化政府廉政建设，政风行风不断改善。

　　政府自身管理不断完善。健全政府绩效考核体系，有力地促进了各项工作完成。加强工程招投标管理，招投标市场秩序进一步规范。对重点项目资金进行跟踪审计，资金使用效益得到提高。优化财力资源配置，保障重点支出能力进一步增强。调整市、区财政管理体制，财政管理水平不断提高。扩大各区管理权限，调动了各区发展积极性。

　　各位代表，过去的一年，是我市实施项目最多、投资力度最大的一年，是经济社会协调发展、人民生活水平不断提高的一年。这些成绩的取得，凝结着全市人民的辛勤汗水，体现着方方面面的通力合作。在此，我代表市政府，向全市各族人民，向各民主党派、工商联、无党派人士、各人民团体和社会各界人士，向中省直单位、驻阜部队、武警官兵和政法干警，向关心支持阜新发展的老领导、老同志，向所有为阜新发展作出贡献的海内外朋友们，表示诚挚的感谢，并致以崇高的敬意！

　　各位代表，由于受严重旱灾影响和金融危机冲击，粮食大幅减产，煤炭、电力等行业增幅回落，直接影响了地区生产总值的增长幅度。同时，我们还看到，经济社会发展中还存在一些突出矛盾和问题。主要有：缺少支撑性的大企业，产业集群规模小、链条短，知名品牌不多，企业竞争力不强；农业产业化、现代化水平不高，农民持续增收能力较弱；财政收支矛盾突出，满足发展需求的压力大；基础设施薄弱，供暖质量不高；棚户区改造遗留问题还没有得到彻底解决；企业融资难，缺乏专业技术和管理人才；安全生产管理仍存在薄弱环节；个别政府部门工作人员工作效率低，办事拖沓，精神状态、思想观念、工作能力还不适应“突破阜新”的迫切要求。对此，我们要高度重视，切实采取有效措施，认真加以解决。

　　二、2010 年工作安排

　　2010 年，是“突破阜新”的关键之年，也是转型发展的跨越之年。当前，我市经济社会发展既面临严峻挑战，更蕴含重大机遇。中央经济工作会议明确提出，继续实施积极的财政政策和适度宽松的货币政策，国家加快实施东北老工业基地振兴、促进资源型城市经济转型及我省实施区域协调发展三大战略，为我市提供了良好的外部环境和政策支持；在国家扩大内需的形势下，我市迎来了高速铁路、高速公路建设高潮，区位优势迅速提升，为我市扩大对外开放、聚集生产要素创造了极为有利的条件；经过多年的建设，我市基础设施日益完善，发展后劲明显增强，具备了大干快上的内外部条件。我们一定要带着强烈的机遇意识、使命意识，满怀发展激情，创造性地开展工作，切实肩负起实现突破发展、推进转型振兴、让全市人民生活更加美好的历史责任。

　　按照市委十届九次全会的部署，今年政府工作的总体要求是：深入贯彻党的十七大和十七届三中、四中全会及中央、省经济工作会议精神，以“突破阜新”为主线，以解放思想为先导，以改革开放为动力，坚持科学发展、创新发展、和谐发展，突出抓好项目建设，扎实推进民生工程，全力保持社会稳定，为实现经济社会又好又快发展而努力奋斗。

　　全市经济社会主要预期目标是：地区生产总值330 亿元，增长20%；全社会固定资产投资400 亿元，增长66.7%；地方财政一般预算收入22 亿元，增长18%；直接利用外资1亿美元，增长56%；城市居民人均可支配收入12400 元，增长11%；农村居民人均纯收入6100元，增长10.2%；万元地区生产总值能耗下降4.5%；城镇人口登记失业率控制在4%以内。

　　（一）集中精力抓好“千个项目大会战”，加快“三大基地”和重点产业集群建设步伐

　　全年实施投资千万元以上项目1000个，其中，新开工项目500个，续建项目500个；竣工投产项目300个。

　　全面推进大唐煤制气项目尽早开工建设，完成投资20亿元。推进巴新铁路项目主体工程完工，做好复线项目前期工作。加快风电项目建设进程，新开工项目20 个，完成投资100亿元，装机容量达到180万千瓦。启动光伏产业发展，开工建设太阳能发电示范项目。大力推进彰武火电项目进程。不断扩大阜新双汇、伊利等企业生产规模，推进鲁花花生油、马铃薯深加工、玉米深加工等项目建成投产。

　　加快培育壮大重点产业集群，着力引进和培育骨干企业和知名品牌，全面推进配套设施和服务体系建设，拉长产业链条，提升产业化水平。建设国家液压装备产业化基地，新开工项目80 个，累计投产项目突破100个。建设现代皮革产业集群，新开工项目80个，富国皮革、德裕制革等20 个项目建成投产。建设中国北方家具研发制造产业集群，新开工项目80 个，累计竣工投产项目40 个。抓住国家特色铸造产业集群试点市机遇，铸造产业集群新开工和竣工投产项目各10个，在发展规模上实现新的跨越。发展优势特色的氟化工产业集群，新开工和竣工项目各20个，在拉长产业链、扩大基地规模上取得新进展。

　　（二）坚持以招商引资为重点，全面提升对外开放水平

　　紧紧抓住省三大区域协调发展战略和我市区位优势提升机遇，加大招商引资力度，促进南方资本投进阜新、产业转移项目聚集阜新，招商引资突破百亿元，增长50%以上。以“三大基地”和产业集群招商为重点，围绕主导产品、上下游配套，针对长三角、珠三角、港台和日韩等重点区域，组织好招商活动。进一步创新招商方式，实施主题概念招商、规划招商、环境招商、产业招商、节会招商和小分队招商等形式，对接洽谈一大批项目。打好经济转型试点市牌，充分利用省直部门帮扶阜新的机制平台，项目、资金、政策“三争取”工作要取得明显成效。加强与中省直企业和500强企业对接，积极引进一批大项目落户阜新。

　　着力优化出口产品结构，进一步做大外贸骨干企业，积极培育新的增长点，实现出口创汇1 亿美元以上。

　　融入沈阳经济区一体化发展，启动沈彰新城10 平方公里起步区的规划建设。依托辽宁沿海经济带开发开放平台，谋划一批与沿海产业相衔接的重大项目，尽快形成腹地与沿海互动格局。加强与蒙东地区的交流，推进基础设施建设、资源开发利用等方面的合作。积极开展对俄经贸合作。

　　（三）坚持走新型工业化道路，提升工业经济整体水平

　　不断提高企业运行质量，从资金、政策、产品研发、市场开拓等方面支持企业发展。新增规模以上工业企业100户。规模以上工业实现增加值96亿元，增长26%。

　　继续实施企业倍增计划，加快培育地方骨干企业和特色品牌，销售收入超亿元企业达到80 户。阜矿集团尽快实现百亿元规模，玄武岩连续纤维一期形成2万吨矿岩棉生产能力，膨润土矿达到60万吨生产能力，开工建设年产300 万吨彰武雷家煤矿。阜新发电公司增加发电量、提高运行效益。阜新供电公司提高电网输送能力和供电水平。

　　加快用高新技术改造传统产业，优化提升产业结构。实施工业技改项目250 项，总投资100 亿元。

　　太克集团10 万吨短流程铸造等30 个亿元以上项目开工建设，大鹰公司4000 吨干法熟料水泥等20 个亿元以上项目竣工投产。制定新兴产业发展规划，重点抓好电子信息、新型材料、生物医药等新兴产业发展。

　　加快培育壮大县区主导产业和特色产业，提升工业在县区经济中的比重，全面完成县区经济三年翻番目标。大力发展民营经济，促进中小企业发展壮大。民营经济增加值增长25%以上。

　　（四）大力发展现代农业，推进社会主义新农村建设

　　加快发展设施农业，新建保护地5 万亩。推进花生产业化进程，花生种植面积达到200万亩。积极发展优势特色产业，培育壮大冰葡萄、马铃薯、树莓等基地，继续推进杂粮、南瓜、食用菌等产业发展。抓好“菜篮子”工程。

　　突出奶业、肉牛、生猪、肉鸡等重点产业发展，不断提高集约化、标准化饲养水平，新建畜禽标准化小区150个。做好重大动物疫病防控工作，保障畜牧业健康发展。

　　促进农村综合改革，积极推动土地承包经营权流转，继续做好集体林权制度配套改革，推动国有林场改革。加快农民专业合作社和专业协会建设，创建专业示范社20个。

　　加强农业基础设施建设，提高农业综合生产能力。加大农村社会事业投入，改善农村生产生活环境。加快以水源工程为重点的水利工程建设，新增灌溉面积3.5万亩，完成4座中型病险水库除险加固主体工程。提高农机装备和作业水平，继续实施百万亩机械化保护性耕作工程。不断提高人工影响天气能力。

　　（五）加快发展现代服务业，努力培育新的经济增长点

　　以高标准规划建设中央商务区为重点，全面提高商业业态水平。加快解放广场服务业集聚区、东市商业区、站前地区建设，启动兴隆百货二期、地下解放广场和商业网点等一批项目，引进零售业品牌，建设星级酒店项目。积极发展社区服务业，抓好养老、医疗、健身、家政等便民项目。

　　加快市场体系建设，市花卉市场及哈尔套、后新秋蔬菜批发市场投入使用，改造建设一批社区标准化菜市场，筹建市再生资源回收加工利用市场。扩大早餐工程、“万村千乡”市场工程和“新网工程”覆盖面。加快北方国际物流园建设，推进“玉龙新城”物流配送中心和交通物流园等项目。开展保税物流区前期工作。

　　坚持统筹规划，实施住宅地产、商业地产、工业地产全面开发，新区建设与老城区更新改造统筹推进，市区与县城同步发展。实现房地产投资40亿元，开工面积400万平方米。加快发展建筑业，产值达到80亿元。

推进海州露天国家矿山公园二期建设，加快打造工业遗产旅游示范区。高水平编制地热产业发展规划，启动建设以“佛、山、泉、湖”为内涵的文化休闲度假温泉城项目。提高海棠山、大清沟等重点景区开发水平，瑞应寺争创国家4A 级景区，高山子风电主题景区全面建成，启动孙家湾历史纪念馆项目。进一步发展民族风情游和农家乐乡村游。

　　以软件动漫、演艺娱乐、广告传媒、印刷出版等为重点，培育文化骨干企业，推进文化产业发展。办好第五届玛瑙博览会，打造特色文化品牌。

　　（六）加强城乡建设和管理，提升城镇化水平

　　发挥规划的龙头作用，认真做好《城市总体规划》修编工作，进一步完善城市功能，打造城市品牌，建设宜居、高效、平安、和谐城市，不断提升城市综合竞争力。

　　加快实施“三位一体”规划，加大市中心区与阜蒙县城连接带的规划建设力度，进一步完善城市空间布局。全面推进“玉龙新城”开发建设，路网框架基本形成，新增开发面积100万平方米。高水平规划建设城市南部地区，提升城市南出口形象。扩建西出口经东梁温泉城到清河门区皮革基地通道，推进阜锦连接带建设。

　　突出抓好基础设施建设。一是推进铁路项目建设。开工建设京沈客专重点控制性工程和沈彰铁路、锦阜铁路扩能项目。二是推进高速公路项目建设。完成彰通、阜盘高速公路一期工程，开工建设阜盘、阜铁高速公路连接工程，加快推进阜奈高速公路前期工作。三是启动军民合用机场项目前期工作。四是实施城市道路畅通工程。加快环城路建设，玉龙西路及南环路竣工通车；继续推进城市主干路的延伸改造，解放大街北段、高孙路、八一路东段、迎宾大街北段、高速公路东站出口路等竣工通车，对中华路进行提升改造。完成国省干道大中修140 公里，彰武火车站及彰武、新邱客运站投入使用。五是实施管网建设改造工程。继续抓好老旧管网改造，供水管网改造全部完成；进一步完善城市供暖设施，提高供暖质量。开工建设锦州至阜新石油管输工程。开展“东水西调”工程前期工作。

　　提高城市管理水平。发扬“人民城市人民建、人民城市人民管”的优良传统，发挥社团、企业、市民在城市建设管理中的积极作用。加强市容环境管理，深入开展专项治理行动，着力解决垃圾乱排放、露天烧烤、机动车违章停放等问题。改善城市道路交通环境，促进交通顺畅。加强城市公交运营管理，调整完善公交线路，更新车辆100台。完善物业管理办法，提高物业管理水平。

　　着力改善城市环境。加快城市水系建设，搞好高林台河、九营子河治理工程，建设玉龙河，提高细河蓄水能力。以“玉龙新城”为重点，集中打造人民大街北段、东新路等城市绿化景观带，加快细河南岸绿化美化，新增城市绿化覆盖面积150 万平方米，争创国家“ 园林城市”。全面开展达标工作，加快创建国家卫生城步伐。

　　统筹城乡协调发展，推进城乡一体化建设，加快县城和中心镇规划建设步伐，加大县、镇（乡）基础设施建设力度，集中打造福兴地、哈尔套等一批特色中心城镇。逐步改革户籍制度，增加城市人口，不断提高城镇化水平。

　　（七）着力改善民生，让发展成果惠及全市人民

　　把扩大就业作为改善民生头等大事来抓。强化就业培训，提高就业能力，促进稳定就业。加强对就业困难群体特别是“零就业家庭”的就业援助，保障大学毕业生充分就业。实现实名制就业5 万人，普惠制培训6 万人，组织城乡劳务输出25万人次。

　　进一步完善社会保障体系。着力推进非公有制经济组织人员、灵活就业人员等基本养老保险参保工作，提高企业退休人员基本养老保险待遇水平。扩大城镇居民基本医疗保险覆盖面。积极开展新型农村社会养老保险试点，提高新农合筹资标准，进一步推进城乡居民医疗救助制度。提高城乡居民最低生活保障和优抚对象保障标准。

　　今年要重点办好十项实事。一是做好棚户区改造和廉租房建设工作；为农村贫困户援建住房1000 套。二是完成50 个老旧小区综合整治，力争改造后小区实现物业管理全覆盖，推广物业公司、业主委员会、社区“三位一体”管理模式。三是进一步完善公园功能，建设一批公共绿地和文化休闲广场。四是新建城市公厕33座，完成城区中小学改厕工作，实施农村改厕2 万座。五是社区“十分钟服务圈”实现全覆盖，农村示范社区达到100 个。六是新建2 所区域性中心敬老院，完善100个社区夕阳红老年图书室建设。七是新建300 所甲级村卫生室；新增标准乡镇文化站13 个、农家书屋320 个、村休闲健身广场38 个。八是实现农村10 万低收入人口稳定脱贫。九是新建“四位一体”生态模式户3000 户和大中型沼气池15座。十是解决6.6万农村人口饮水安全问题。

　　（八）做好金融财政工作，提高资金保障能力

　　创新投融资体制，打造多层次的融资平台，为城市发展提供资金保障。全年新增贷款突破100 亿元。阜新银行完成增资扩股，沈阳分行正式开业。激活民间资本，新增小额贷款公司20家，新建村镇银行2 家，各县区均成立担保公司。规划建设开发区金融服务中心。培育壮大金融市场，力争引进1 家域外金融机构。城投债一期发行，大金公司争取上市。运用BT、BOT 等多种方式，提高市场化融资能力。支持保险业快速发展，发挥其保障民生、服务经济社会发展的功能。

　　巩固提高现有税源，培育壮大新兴税源，挖掘税收增长潜力。整合各类财政资金，优化财力配置，带动社会投资，推动项目建设。加强税收监管，强化对采矿业、房地产业、餐饮娱乐、交通运输等重点领域税收征管。继续落实非税收入管理各项政策措施，强化土地、矿产等国有资源有偿使用收入征缴。树立过紧日子思想，严格控制一般性支出，建设节约型机关。深化财政改革，逐步健全公用经费定额管理体系，对非税收入实行指标管理，扩大国库集中支付范围。

　　加强国有资产监管，对正常生产经营的国有企业实行目标绩效考核。继续推进国有企业改革，启动乌龙、伊马煤矿政策性破产工作，妥善处理国有企业历史遗留问题。

　　（九）加强生态建设和环境保护，增强可持续发展能力

　　加快林业生态建设，重点推进荒山治沙造林、村屯绿化等林业工程建设，人工造林和封山育林77万亩。着力打造全国最大的樟子松育苗基地，章古台自然保护区晋升国家级自然保护区。巩固封山禁牧成果，坚决遏制毁林开荒行为。继续抓好河道生态和水土生态保持工程建设，加快海州露天矿、高德矿和东梁矿环境治理，辽西危险废弃物处置中心主体完工。推进资源型城市生态恢复试点市建设取得积极进展。

　　提高水资源开发、利用、管理水平，合理配置水资源，促进水资源循环利用。加强水环境整治，保护地下水。清理盘活闲置、低效利用土地，提高土地投资强度、容积率和建设密度，促进土地节约集约利用。

　　落实节能减排责任制，做好高耗能企业的节能降耗工作；加大烟尘整治力度，全部取缔城市中心区10 吨以下燃煤锅炉，提高城市空气质量。二氧化硫、化学需氧量排放分别下降1.6%和20.1%。推行清洁生产，发展低碳经济，开工建设一批循环经济项目，加快循环经济试点市建设步伐。

　　（十）积极发展各项社会事业，推进和谐阜新建设

　　加快科技创新体系建设，加强与高校和科研院所对接，促进产学研合作。开发高新技术产品60个，高新技术产品增加值突破10 亿元。深入实施人才兴市战略，充分发挥辽工大人才、技术优势，继续做好硕士研究生和博士后科研基地的人才引进工作，积极培养适应发展的各类人才。

　　深入推进新一轮“普九”，加强教育教学改革、教师培训工作，不断改善农村师资条件，促进义务教育均衡发展。规范发展学前教育，引导、支持民办教育发展。大力发展职业教育，辽工大职业技术学院新校区投入使用，加快二职专新校建设进程。以阜新高专为依托，加快发展地方高等教育。新建塑胶操场28 块，城区中小学操场全部改造完成，适时启动县城中小学“ 操场工程”。积极推进中小学校舍安全工程。新建农村九年制学校4所。

　　繁荣文化事业，提高城市文化软实力。组织开展丰富多彩的群众性文化活动，举办首届社区文化节和职工文化技术节。促进文化创作，打造文艺精品，积极备战省艺术节。市图书馆、数字电视服务平台投入使用，开工建设市档案馆、博物馆。启动市广播电视大厦、群众文化艺术中心项目。全面建设村级文化广场，力争每个行政村都有一个文化活动场所。

　　以建设全国一流篮球城为重点，广泛开展全民健身运动。加强体育后备人才基地建设，提高竞技体育水平，篮球等重点项目在省运会上争创佳绩。市体育馆投入使用，新建篮球广场3处，启动市民健身馆项目。

　　大力发展医疗卫生事业。加强公共卫生应急体系建设，提高重大疾病防控能力。推进医疗卫生体制改革，加强医德医风建设，提高医疗服务水平。进一步改善医疗卫生条件，市中心医院病房楼、阜矿总医院急救中心投入使用，开工建设第四人民医院病房楼。深入开展减盐行动，普及健康知识，积极倡导健康生活方式。继续做好人口计划生育工作，提高出生人口素质。市新殡仪馆投入使用。

　　进一步支持蒙古族文化事业及蒙医、蒙药等民族产业发展，不断巩固发展民族团结进步事业。依法加强管理，发挥宗教在促进经济发展、社会和谐中的积极作用。

　　加强民主法制建设。自觉接受人大的依法监督和政协的民主监督，认真办理人大代表议案、建议和政协提案。加强与各民主党派、工商联、无党派人士的联系。支持工会、共青团、妇联等组织开展工作。支持法院、检察院依法履行职责。进一步巩固军政军民团结，继续推进民兵预备役建设。加强基层政权建设，组织好第十届村委会换届选举工作。扎实开展好“ 五五”普法活动。高水平编制完成“ 十二五”规划。全面实施县区在地统计工作。

　　推进“ 友爱阜新”建设。把社会主义核心价值体系贯穿于精神文明建设全过程，用社会主义荣辱观引领风尚。加强公民思想道德建设，提高城市文明程度，倡导热爱家乡、赞美阜新的社会新风。关心残疾人事业。发展社会福利和慈善事业。

　　推进“ 平安阜新”建设。加强应急管理体系建设，有效预防预警和处置各类突发事件。完善社会矛盾大排查、大调解工作机制，积极化解各类社会矛盾。层层落实安全生产责任制，坚决遏制重特大事故发生。强化食品药品源头监控和市场监管，保障食品药品安全。完善社会治安防控体系，加强社会治安综合治理，依法防范和打击违法犯罪行为，维护社会和谐稳定。

　　（十一）不断学习创新，努力建设勤政廉洁、高效务实的服务型政府

大力推进政府管理创新、工作方式改进和作风转变，不断提高府服务人民、推动发展的能力。

　　第一，保持昂扬的发展激情，创造性地开展工作。要做学习创新型干部，建设学习型机关。以世界的眼光、战略的思维、本土化行动，高标准做好本职工作。把解放思想贯穿工作始终，建设有利于创新的制度环境，加大体制机制创新力度。推进政府工作实行项目化管理，建立健全目标、责任、考核体系，提高政府工作效率和管理水平。强化对政府系统干部队伍的教育、管理和监督，努力建设一支业务精通、作风过硬、人民满意的干部队伍。各级干部要始终保持一股迎难而上的勇气、一股敢为人先的锐气、一股干不上去誓不罢休的志气，在全市上下形成干事创业求突破、争先创优促发展的浓厚氛围。

　　第二，进一步提高工作效能，不断增强服务能力。简化行政审批程序，扩大全程代理和并联审批范围，推行上门服务、预约服务制度。提高电子政务网络平台应用水平，促进政府工作方式转变。加大行政问责力度，加强行政效能监察、审计监督和财政监督，实施重点工程、重大项目、重要资金跟踪问效。深化政府绩效考核机制，增强考核结果的导向性、激励性和鞭策性。大兴求真务实、真抓实干之风，坚持深入一线，调查研究，现场办公，重实干、办实事、求实效。

　　第三，始终坚持执政为民，密切联系群众。把实现好、维护好、发展好人民群众的根本利益作为一切工作的出发点和落脚点，用心倾听人民群众的意见，满腔热情为人民群众办好事、解难题。全面推进政务公开，保障市民知情权、参与权、表达权和监督权。发挥好市长公开电话、市长信箱、行风热线作用，进一步畅通民意渠道。采取有力措施，努力增加城乡居民收入，提高群众生活质量。坚持问政于民、问需于民、问计于民，促进政府决策民主化、科学化，使政府各项工作体现群众意愿、符合群众利益、满足群众需求。

　　第四，积极推进依法行政，大力加强廉政建设。全面落实国务院《关于加强市县政府依法行政的决定》，努力提高行政机关及其工作人员依法行政的能力和水平。扎实推进惩治和预防腐败体系建设，严格执行党风廉政建设责任制，强化领导干部廉洁自律。严肃查处滥用职权、贪污腐化、失职渎职、商业贿赂和严重损害人民群众利益的案件。各级领导干部要带头勤政廉政，以实际行动取信于民，树立风清气正的良好形象。

　　各位代表，“突破阜新”的使命激励我们团结奋进，人民群众的重托不容我们丝毫懈怠。任何挑战和困难都阻挡不了阜新人民突破发展的坚定步伐。让我们在市委的正确领导下，紧紧依靠全市各族人民，锐意进取，开拓创新，在阜新转型振兴的道路上奋勇前进！
